# Uni- and triaxial accelerometric signals agree during daily routine, but show differences between sports

**DOI:** 10.1038/s41598-018-33288-z

**Published:** 2018-10-10

**Authors:** Maia P. Smith, Alexander Horsch, Marie Standl, Joachim Heinrich, Holger Schulz

**Affiliations:** 1 Institute of Epidemiology, Helmholtz Zentrum München - German Research Center for Environmental Health, Neuherberg, Germany; 2 Department of Computer Science, UiT The Arctic University of Norway, Tromsø, Norway; 3 Comprehensive Pneumology Center Munich, Member of German Center for Lung Research (DZL), Munich, Germany; 4 Institute and Outpatient Clinic for Occupational, Social and Environmental Medicine, Inner City Clinic, University Hospital of Munich (LMU), Munich, Germany; 5 Department of Public Health, School of Medicine, St George’s University, True Blue, Grenada

## Abstract

Accelerometers objectively monitor physical activity, and ongoing research suggests they can also detect patterns of body movement. However, different types of signal (uniaxial, captured by older studies, vs. the newer triaxial) and or/device (validated Actigraph used by older studies, vs. others) may lead to incomparability of results from different time periods. Standardization is desirable. We establish whether uniaxial signals adequately monitor routine activity, and whether triaxial accelerometry can detect sport-specific variations in movement pattern. 1402 adolescents wore triaxial Actigraphs (GT3X) for one week and diaried sport. Uni- and triaxial counts per minute were compared across the week and between over 30 different sports. Across the whole recording period 95% of variance in triaxial counts was explained by the vertical axis (5th percentile for R^2^, 91%). Sport made up a small fraction of daily routine, but differences were visible: even when total acceleration was comparable, little was vertical in horizontal movements, such as ice skating (uniaxial counts 41% of triaxial) compared to complex movements (taekwondo, 55%) or ambulation (soccer, 69%). Triaxial accelerometry captured differences in movement pattern between sports, but so little time was spent in sport that, across the whole day, uni- and triaxial signals correlated closely. This indicates that, with certain limitations, uniaxial accelerometric measures of routine activity from older studies can be feasibly compared to triaxial measures from newer studies. Comparison of new studies based on raw accelerations to older studies based on proprietary devices and measures (epochs, counts) will require additional efforts which are not addressed in this paper.

## Introduction

Physical activity (PA) is a major protective factor for most noncommunicable diseases^1,2^ and it is generally accepted that most populations in the developed world are insufficiently active^3^. However, estimates of PA levels and time trends vary: in many developed countries neither cross-sectional levels^4^ nor size and direction of time trends^5–7^ have been established. Thus associations are difficult to establish and interventions are difficult to design.

Because accelerometry is scalable and objective, it is a popular technique for assessing PA under field conditions. However, accelerometry has its own limitations: perhaps most obviously, acceleration is only an indicator of PA, and accelerometers register more movement (counts) during some activities (e.g. walking) than others (e.g. cycling)^8,9^. The earliest devices (pedometers) were only intended to monitor ambulation; they were succeeded by uniaxial accelerometers, which measure all acceleration in the vertical axis; and then triaxial accelerometers, which measure all acceleration in all three axes. These are currently the research standard for capturing acceleration caused by body movement^10,11^, particularly when assessing complex movements: however, some research also uses consumer-grade wearable devices such as smartwatches^12^ and mobile-phone accelerometers^13–15^, which are less expensive and more accessible than dedicated accelerometers^14^. Each new generation of devices represents an advance in precision and/or cost over the previous generation.

However, PA estimates are more useful if they are directly comparable with earlier studies. Longitudinal studies of PA often rely on self-reported activity at the earlier timepoint, and changes in the popularity of different activity domains (e.g. occupational PA vs. leisure PA)^5,6,16^ make these reports difficult to compare: indeed, there is no scientific consensus regarding changes in total PA in the US^5^ over the past few decades. This issue may be resolved by longitudinal accelerometric measures: accelerometry is indifferent both to the domain in which^13–15^ activity takes place, and to reporting bias^17^. It is also objective, correlates well with energy expenditure^10,18^, and is rapidly becoming more scalable as prices drop. To make the best use of this objectivity it is important to make estimates of accelerometric PA back-compatible, which in turn may mean minimizing variation due to factors not of primary interest. In addition to data-handling protocols and site of wear (e.g. hip^19^, wrist^12^, ankle) one of these may be the number of movement axes captured by the device or considered in data handling. Uniaxial and triaxial counts have been shown to correlate well during daily living^20^ and to have similar relationships with total energy expenditure^11^; so if uniaxial accelerometry is comparable to triaxial, for some applications the added precision of a triaxial signal may be outweighed by the concurrent lack of comparability between earlier and later studies.

Similar reasoning applies to the choice of the validated Actigraph device rather than, or in addition to, newer devices such as smartphone accelerometers^13–15^. While these devices are objective, apparently valid^15^, and readily available, differences in data-handling protocols may create differences in estimated activity that are at least as large as true population differences. (This has been shown to be the case for different protocols even within the Actigraph)^21^. Activity estimates from one device may thus be comparable only to others from that device, or even that data-handling protocol (e.g. app version): a situation which makes studies difficult to compare and time trends in activity impossible to establish. Thus if the current study shows that the Actigraph is adequate to capture sport-specific movement patterns, it will support the continued use of this device for back-compatibility wherever financially and practically possible. Conversely, if it cannot capture these patterns then fragmentation of research may be inevitable until another standard device is found to replace it.

In this study we estimate the magnitude of differences between the uniaxial and triaxial accelerometric signal under field conditions in a cohort of 1402 adolescents who wore the validated Actigraph device for one week of daily routine, including sport. Since one major posited benefit of triaxial accelerometry is its ability to monitor complex patterns of body movement^11^, we calculate the ratio between triaxial and uniaxial (vertical-axis) counts during daily living, and then during over 30 different sports, including dancing, rowing and jogging. Differences are likely to be largest between these sports, and thus the additional benefits of triaxial devices are most visible.

## Methods

This study sampled adolescents from two different population-based German birth cohorts: GINIplus and LISAplus, born between 1995 and 1999 in the regions of Munich and Wesel. Accelerometry was done between 2011 and 2014, and subjects were 15.6 (SD 0.5) years old at the time of accelerometry. Details on study design and cohort selection are published elsewhere^22–24^. Both studies were approved by local Ethics Committees (Ethics Committees of Bavaria and West-Rhine Westphalia) and received written informed consent from all participants and their families. No experiments were performed. All data were collected in accordance with relevant guidelines and regulations.

Accelerometry participants were recruited from the entire 15-year followup of GINIplus and LISAplus that lived in Munich and Wesel, which is all of GINIplus but only 64% of LISAplus. Further details on followup have been previously published^24,25^. Of the 3199 subjects from GINIplus who were successfully recontacted at age 15, all were approached for accelerometry, 1890 (59%) gave initial consent and 1247 (66%) gave final consent, completed successfully, and returned the device. Of 1107 LISAplus subjects who were from Munich or Wesel and thus approached for accelerometry, 654 (59%) gave initial consent and 435 completed (66%). Of the 1682 adolescents from GINIplus and LISAplus who completed accelerometry, 1411 (83%) successfully passed data-quality checks and 1402 wore a device that captured triaxial acceleration. These 1402 are included in the current study.

### Accelerometry Protocol: Overview

Accelerometry protocol has been previously described^25^. Briefly, triaxial accelerometers (ActiGraph GT3X, Pensacola, Florida) were worn on the dominant hip for up to 7 days, after which they were returned by mail. An activity diary was kept throughout, and data were validated against it using automatic and manual methods.

### Activity Diary

Subjects were instructed to document each of the following events as close as possible to the time they occurred: time of waking up and going to bed; time and reason for removing the monitor (non-wear time) such as for showering or swimming; time and method of travel to and from school, such as by walking or driving; time of starting and finishing school; time of starting and finishing school sport; and time and type of leisure sporting activity. Sample diary has been previously published^25,26^.

### Data Management and Quality Control

Sampling rate was set to 30 Hz and the measured accelerations stored at 1 Hz after conversion into activity counts. Counts were summed over 60-second epochs. Data filtering was set to default (‘normal’) as recommended by ActiGraph. Activity counts of all three axes (vertical, horizontal and mediolateral) were measured. ActiLife software was used for initialization of accelerometers (version 5.5.5, firmware 4.4.0) and for download of data. PA data were checked to identify invalid days both by visual inspection and by semiautomatic methods. Diary information was digitized using a 7-day template and a specific coding for events such as sickness, trips, type of sport performed, and non-wear time (NWT). Data entries were reviewed by a second study assistant to avoid transcription errors.

### Validation of wear time

Sensor non-wear time (NWT) was identified both by visual inspection of accelerometer tracings and by comparing the diary data to the results from the monitor using SAS programs published by NHANES^27^. These programs identify probable NWT as at least 60 minutes of consecutive zero counts with less than two consecutive intervals with counts less than or equal to 100. In most cases the diary agreed with the automatic programs upon wear time and NWT.

Of a total 11,572 recorded days in the study cohort, 2740 (17.1%) were invalid^25^. Most invalid days (1140, 58%) were the result of inconsistency between the diary and the NHANES weartime criteria, reflecting our high standard of data cleaning and suggesting a relatively accurate allocation of activity on the days that passed quality control. Other reasons included non-wear time issues (526 days, 26.7%), and technical issues (145 days, 7.4%). Many days were invalid for more than one reason.

### Validation of days

Since our goal was to measure typical activity, subjects were required to have at least one valid weekend day of recording in addition to at least three valid weekdays.

Days were required to have at least 10 hours of valid recording time to be considered valid, or as little as 7 hours if subjects were awake for less than 10 hours, as is recommended elsewhere^25,26^.

### Statistical Methods

All statistical analyses used SAS 9.2. All graphics were created using Excel. All data were limited to validated recording time where the subject reported being awake and out of bed.

Differences and similarities between uniaxial and triaxial counts were expressed first as correlation, then as rank correlation, and then as ratio. Correlations are presented as an indicator of how much additional information the triaxial signal provided over the uniaxial (how well one could be used to predict the other); ratio is presented as an indicator of how much the pattern of movement (percentage of total acceleration that was vertical) varied by activity.

Pearson correlation between uniaxial and triaxial counts minute-by-minute was calculated during all wear time for each subject, and expressed as % of variance explained. Although strict model assumptions (e.g. normality of errors) were often not met, Pearson correlation has benefits including the ability to be meaningfully averaged; easy interpretability as % variance explained; and a greater susceptibility to the effects of extreme values, which makes it a conservative measure of how much information in one measure is captured by the other (i.e. it tends to underestimate the strength of the relationship). Thus we present it in addition to the distribution-independent Spearman’s rank.

Ratio between uniaxial and triaxial counts was calculated for each minute of wear, and then averaged either by subject or by sport. When ratio was presented by sport, we present data only from those sports that were performed at least 10 times and by at least 5 subjects. In this plot the mean and standard error of this ratio is plotted against the mean of uni- and triaxial counts, similar to a Bland-Altman plot^28^.

## Results

Daily activity for 1402 Germans (mean age 15.6, 46% male) was accelerometrically monitored over 4–7 days per subject for an average of 14.7 hours per day. For the average subject, uniaxial counts explained 95% of the variance in triaxial counts (Table 1) and the 5^th^ percentile for this correlation was 91%: in 95% of subjects, the correlation was 91% or higher. Results were similar for rank correlation.

**Table 1:** Population Characteristics.

Mean (standard deviation) unless otherwise stated	
N	1402
Male (N, %)	650, 46
Age, years	15.6 (0.5)
Height, cm	172 (8.2)
Weight, kg	61.6 (11)
BMI, kg/m^2^	20.8 (3.0)
Parents highly educated^a^, %	71
From Munich rather than Wesel, %	61
Reported time in sport, min/day	26.4 (32)
Days of accelerometry (range 4–7)	6.26 (0.88)
Accelerometric min/day	884 (51)
Uniaxial counts/min Mean (SD); 5^th^, 95^th^ percentiles	354 (143) 191, 586
Percent of variance explained between uniaxial and triaxial counts^a%^, Mean (SD); 5^th^, 95^th^ percentiles	94.6 (1.9); 91.3, 97.2
Squared rank correlation between uniaxial and triaxial counts^c^ Mean (SD); 5^th^, 95^th^ percentiles	92.9 (8.6) 76.3, 99.5
Ratio: uniaxial/triaxial, % Mean (SD); 5^th^, 95^th^ percentiles	31.3 (5.8) 22.6, 41.6

^a^Higher-educated parent entered college or higher. Very similar population profiled in (Smith *et al*., Plos One, 2016; 10.1371/journal.pone.0152217).

^b^Within-subject R^2^, expressed as %.

^c^Within-subject Spearman’s rank correlation, squared for comparability with Pearson.

**Figure 1:**
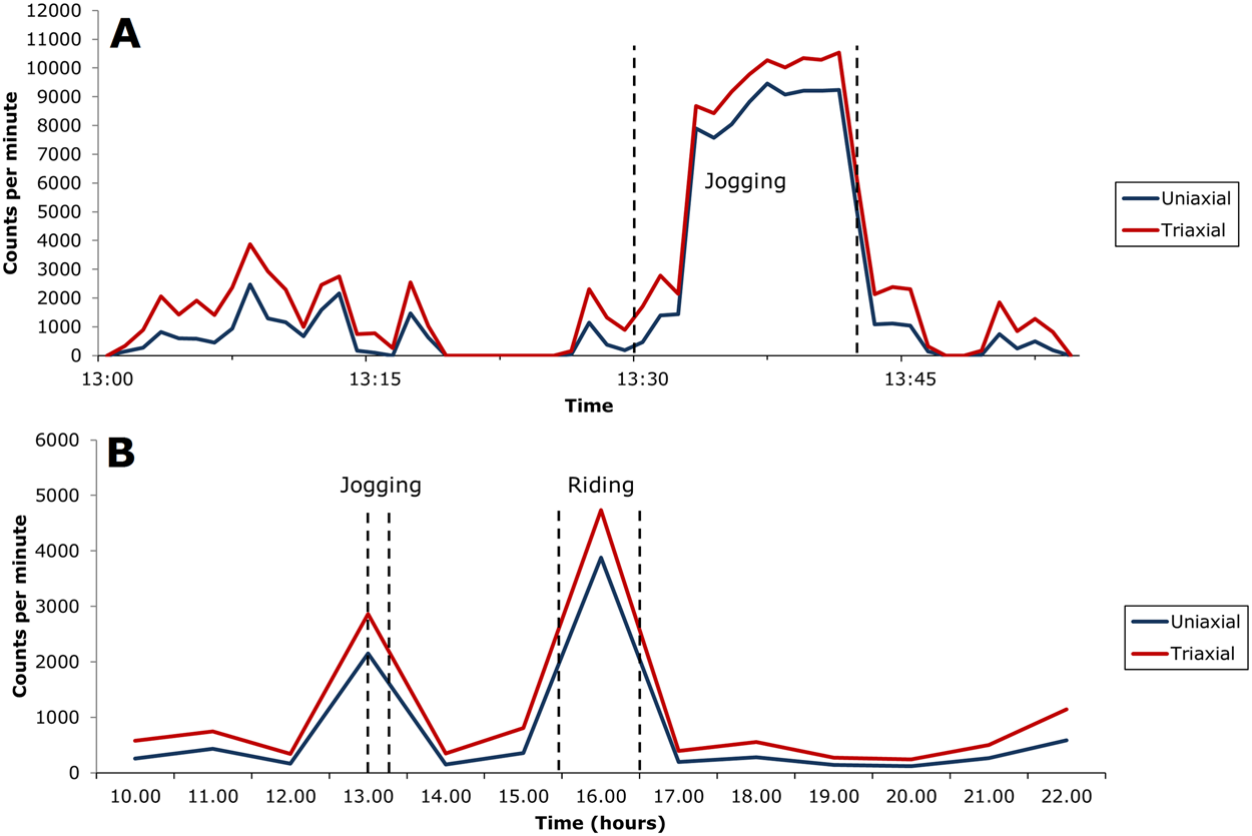
Vertical-axis (uniaxial) and triaxial accelerometric counts averaged by minute (**A**) and hour (**B**) during validated accelerometer wear time. Sport names and times from activity diary.

**Figure 2:**
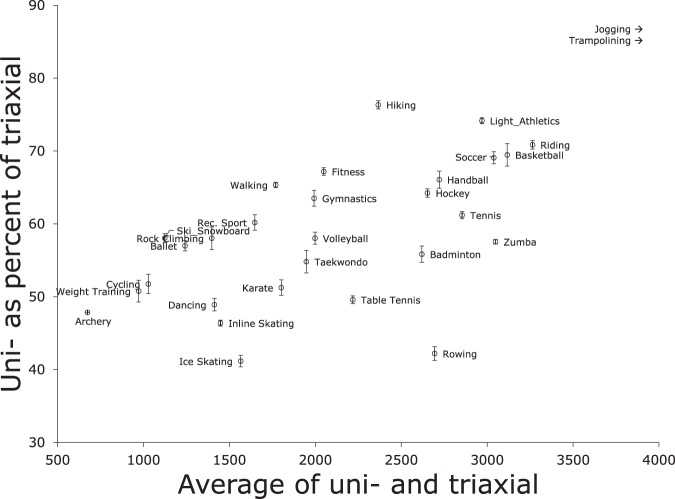
Mean ratio, expressed as percentage, of vertical-axis (uniaxial) to triaxial accelerometric counts during diaried sporting time. Data available in Table 2. Error bars for 1.96 standard errors. Sport names and times from activity diary. Sports shown only if done at least 10 times in our sample, and done by at least 5 subjects. For space, jogging and trampolining not shown.

In an average minute during daily routine, the ratio between uni- and triaxial counts was 31% (SD 6%); 5^th^ and 95^th^ percentiles were 23% and 42%. As activity intensity increased the average ratio rose: that is, a larger fraction of total acceleration was in the vertical axis, increasing to almost 100% when total acceleration reached 6,000 counts per minute. (Fig. 1) However, for a given acceleration level differences between sports were visible: horizontal movements such as rowing tended to have a lower ratio, complex movements such as tennis were intermediate, and vertical movements such as jogging had the highest ratio (Table 2, Fig. 2).

**Table 2:** Comparison of Uni- and Triaxial Counts by Sport.

Sport Name from activity diary	Accelerometric counts per minute (mean)			Ratio (uni -/triaxial) %
	Uniaxial	Triaxial	Mean (uni- and triaxial)	
Drumming	241.1	678.6	459.9	36
Ice Skating	913.3	2219	1566	41
Rowing	1599	3790	2695	42
Yoga	274.4	643	458.7	43
Inline Skating	917.8	1979	1448	46
Archery	436	911.3	673.7	48
Dancing	927.9	1897	1412	49
Table Tennis	1471	2966	2219	50
Karate	1221	2383	1802	51
Weight Training	654.4	1289	971.7	51
Cycling	701.2	1355	1028	52
Taekwondo	1379	2516	1948	55
Badminton	1878	3363	2621	56
Ballet	901.2	1581	1241	57
Rock Climbing	1026	1769	1398	58
Ski/Snowboard	825.5	1422	1124	58
Volleyball	1467	2529	1998	58
Rec. Sport	1239	2058	1649	60
Tennis	2167	3541	2854	61
Vaulting	1511	2395	1953	63
Gymnastics	1548	2437	1993	64
Hockey	2075	3230	2653	64
Walking	1398	2140	1769	65
Handball	2165	3277	2721	66
Fitness	1647	2451	2049	67
Basketball	2555	3678	3117	69
Soccer	2483	3595	3039	69
Riding	2707	3821	3264	71
Light Athletics	2529	3410	2970	74
Hiking	2049	2684	2367	76
Trampolining	5947	7027	6487	85
Jogging	5883	6540	6212	90

Figure 1 (“Uniaxial and triaxial counts”) shows uniaxial and triaxial counts over time for a randomly selected subject, first minute-by-minute over one hour (Fig. 1A) and then averaged by hour over that same day (Fig. 1B). The dotted lines indicate periods of sport, in this case mostly ambulatory sport (jogging in Fig. 1A, jogging and horseback riding in Fig. 1B) with correspondingly high acceleration. Correlation between the two signal types is close, but the ratio depends on total acceleration. During periods of high acceleration, almost all counts are in the vertical axis while this is less true during periods of low acceleration.

Figure 1 shows that averaged across the whole day, the ratio between uni- and triaxial counts increased as activity intensity increased. This was also true during sports, both minute by minute (Fig. 1) and averaged across subjects by sport (Table 2, Fig. 2, “Differences between uniaxial and triaxial accelerometric monitoring of sports”; for space, the high-acceleration jogging and trampolining are not shown). However, when total acceleration was comparable the ratio varied by type of body movement, with ambulation having the highest ratio, complex movements intermediate, and horizontal movements the lowest (Fig. 2).

For example, the mean of triaxial and uniaxial counts (total acceleration) was similar for rowing, badminton, handball, and hiking: however the ratio was not. This reflects the different movement patterns of the sports. Average acceleration for all four was between 2000 and 3000 counts per minute, with hiking less than rowing (2367 counts per minute for hiking, compared with 2695 for rowing). However, during rowing uniaxial counts were only 42% of triaxial (Table 2) reflecting the smooth horizontal movement of the sport. The ratio rose to 56 and 66% for badminton and handball, and was 76% during the purely ambulatory hiking. Likewise, the ratio was 41% for ice skating, 60% for general recreational sport, and 65% for walking although all three had very similar acceleration levels (mean range 1566–1769 counts/min).

Both uni- and triaxial accelerometric monitoring of low-acceleration sports such as cycling and weight training was quite low, reflecting a likely underestimation of energy expenditure. In both of these, the mean of uni- and triaxial counts was about 1,000 counts/min: less than during walking.

## Discussion

This study is among the first to empirically compare uni- and triaxial accelerometric monitoring of different sports under field conditions, using the validated Actigraph device, while simultaneously comparing monitoring of daily routine. We concur^9,11,20^ that different sports are differently monitored by the two signal types, with the ratio of uniaxial to triaxial counts ranging by a factor of 2 between sports consisting of differing amounts of vertical and horizontal movement of the torso, even when total acceleration was comparable. Triaxial accelerometric signals may be a significant improvement over uniaxial during horizontal or complex movements, especially if the goal of the study is to identify health-relevant behaviors by evaluating body movement pattern.

By confirming that the validated Actigraph device was able to capture differences in body movement pattern^29^ we suggest that Actigraphs may yield adequate data for the current generation of research which identifies specific behaviors^29^. In addition to the tendency for total triaxial acceleration to be more vertical (higher ratio) under conditions of higher acceleration, which may be partly the result of artefact such as the known tendency for ActiGraph’s frequency-dependent filtering to attenuate and decrease counts at high intensity, sports with different movement patterns had different ratios between uni- and triaxial counts. Differences between activities would likely be even larger and clearer if more sophisticated techniques for data handling techniques were used: we encourage future researchers to explore and publish such techniques for detecting specific activities with the Actigraph.

While triaxial accelerometry outperformed uniaxial accelerometry in capturing accelerations of sports consisting of smooth, horizontal or complex body movements, such as cycling and skating^9^, it still apparently undermonitored activity during these sports. For example, although jogging and cycling have similar metabolic demands^30^ an accelerometer registered 8 and 5 times as many uni- and triaxial counts during jogging as during cycling; uniaxial accelerometry recorded 74% of jogging time but only 6% of cycling time as moderate-to-vigorous physical activity in this cohort^8^. Both signals also registered low activity during high-energy, low-acceleration sports such as weight training, and previous research^31^ has found similar close relationships between uni- and triaxial monitoring of ambulation. Altogether we confirm that regardless of the number of axes captured by accelerometry, it measures only acceleration and not either physical activity or energy expenditure^18^ and thus may be of limited value when assessing energy expenditure during low-acceleration sports such as cycling or comparing the intensities of activities with different patterns of body movement. In populations where low-acceleration sports are significant contributors to total activity, accelerometry may not be a good choice of metric: other metrics, such as heart rate monitors^10,32^, may be more accurate.

While differences between sports were visible, we also confirm earlier findings which found close correlation between uniaxial and triaxial accelerometric counts both during activities of daily living^11,20,25,31,33^ and, under laboratory conditions, during ambulation specifically^31^. Thus if the goal is to capture daily routine in a cohort where low-acceleration sport makes up only a small fraction of total recording time, differences in uni- and triaxial monitoring may have negligible effects on estimated total activity^33^. This is likely to be the case for our population, in which sports accounted for less than 4% of recording time^25^ although more than two-thirds of subjects participated in sport during the recording week. Indeed, it has been previously shown by us and others that differences between accelerometric estimates of total activity attributable to data handling, such as epoch length^34^, device weartime, and/or cutpoints between activity intensities^4,21^, are at least as large as those solely attributable to the difference between a uni- and a triaxial signal^17,35^. Thus although differences between uni- and triaxial accelerometry are apparent for specific sports, if the goal is to monitor daily routine (as it often is)^10,36^ it appears feasible to compare newer studies using triaxial accelerometry with older studies which relied on uniaxial accelerometry^33,37^.

There is an ongoing trend in the physical activity community to harmonize research by indicating activity with raw triaxial accelerometric data^38^ rather than proprietary measures (epochs, counts) which are often device-specific. Doing so will enable longitudinal studies over several generations of devices and the comparison of estimates from different timepoints, but it will require the creation of models to estimate proprietary older measurements from raw acceleration data.
